# Botulism and Preserved Green Olives

**DOI:** 10.3201/eid1105.041088

**Published:** 2005-05

**Authors:** Amy Cawthorne, Lucia Pastore Celentano, Fortunato D'Ancona, Antonino Bella, Marco Massari, Fabrizio Anniballi, Lucia Fenicia, Paolo Aureli, Stefania Salmaso

**Affiliations:** * European Programme for Intervention Epidemiology Training, Rome, Italy;; † Istituto Superiore di Sanità, Rome, Italy

**Keywords:** botulism, outbreak, green olives, Italy

**To the Editor:** In March 2004, a total of 16 suspected cases of botulism were reported to the Italian National Institute of Health by hospitals in 3 adjoining regions in central and southern Italy (Molise, Campania, and Puglia). Initial investigation showed that all patients had eaten at the same restaurant in Molise on February 22 or 24, 2004. The restaurant provided reservation lists for those dates (the restaurant was closed on February 23). It also provided a list of foods that had been served each evening. Persons on the reservation lists were contacted and asked to provide the names of others who had been at their tables to ensure that as many diners as possible were traced. Of 73 persons who had been identified as having eaten at the restaurant on either evening, 66 were successfully contacted and interviewed in person or by telephone about symptoms and food consumed at the restaurant.

For purposes of the investigation, a probable case-patient was defined as a person who had dined at the restaurant on February 22 or 24 and had experienced diplopia or blurred vision and at least 1 of the following symptoms: dysphagia, dry mouth, dysarthria, upper/lower extremity weakness, dyspnea, and severe constipation. Those who met the probable case definition and had laboratory-confirmed botulism were considered definite case-patients.

We tested for botulinum neurotoxin in serum and spores in stool samples as described (1). Serum or stool specimens from 24 patients with ≥2 symptoms were sent to the Italian National Institute of Health for testing.

Twenty-eight persons reported ≥2 symptoms (42% attack rate); 25 (89%) were considered probable cases and 3 (11%) were considered confirmed cases. Two members of the restaurant owner's family and 1 employee were among the probable case-patients. Onset of symptoms occurred 4 hours to 6.5 days after eating at the restaurant (median 36 hours). Twenty persons (71%) had been seen in emergency rooms, 15 (53%) were admitted to a hospital, and 18% were admitted to intensive care. None required ventilatory support, and no deaths occurred.

The main symptoms reported by 28 probable and confirmed patients included dry mouth in 25 (89%), dysphagia in 25 (89%), severe constipation in 22 (79%), and blurred vision in 27 (96%). Three weeks after onset of symptoms, 15 (68%) reporting severe constipation, 11 (41%) reporting blurred vision, 10 (40%) reporting dry mouth, and 11 (44%) reporting dysphagia still had these symptoms. Of the 24 patients for whom rectal swabs were available, 3 were culture-positive for *Clostridium botulinum* type B. None of 5 serum samples tested positive.

Food-specific attack rates, relative risks (RRs), and 95% confidence intervals (CIs) were calculated. A Poisson model with robust error variance was used to estimate RR with adjustments for possible confounding and effect modification (2). Foods associated with illness with p values <0.20 were considered in the model.

In a univariate analysis in which all 28 patients were considered, the RR of illness was higher among diners who ate home-preserved green olives in salt water (RR 5.2, 95% CI 1.4–19.8), ate cream pastries (RR 2.5, 95% CI 1.8–3.4), and drank homemade lemon liqueur (RR 2.1, 95% CI 1.3–3.4). After multivariate analysis, only the risk associated with eating green olives remained significant (RR 5.2, 95% CI 1.4–19.8).

None of the food items served on February 22 or 24 was available for sampling, and none of the other 13 food samples obtained from the restaurant was positive for *C. botulinum*. However, the pH of a jar of olives that had been prepared at the same time as those eaten on February 22 and 24 was 6.2, far above the level of 4.6 required to prevent growth of *C. botulinum*. No salinity testing was performed by the local laboratory, and inadequate storage during transit made it impossible to conduct salinity and water activity tests at the national reference laboratory.

Interviews with the restaurant proprietors indicated that the olives were prepared on site during the fall of 2003 from local olives. After soaking in salt water for ≈35 days, the olives had been decanted into jars, and salt water had been replaced with fresh water. Neither the amount of salt used in the salt water mixture nor the pH at any stage was standardized during preparation.

Both epidemiologic evidence and information obtained regarding preparation of the olives strongly suggest that they were the likely source of the outbreak. This outbreak highlights the previously documented risk associated with improperly prepared olives (3–5). In Italy and elsewhere in Europe, an increasing trend favors traditional foods and preparation methods over large-scale industrial products. This outbreak underlines the importance of providing training and periodic monitoring of those involved in small-scale preparation to ensure that disease risks from improperly prepared or stored foods are minimized.
